# Distinct Spontaneous Brain Activity Patterns in Different Biologically-Defined Alzheimer’s Disease Cognitive Stage: A Preliminary Study

**DOI:** 10.3389/fnagi.2019.00350

**Published:** 2019-12-17

**Authors:** Qingze Zeng, Xiao Luo, Kaicheng Li, Shuyue Wang, Ruiting Zhang, Hui Hong, Peiyu Huang, Yeerfan Jiaerken, Xiaojun Xu, Jingjing Xu, Chao Wang, Jiong Zhou, Minming Zhang

**Affiliations:** ^1^Department of Radiology, The Second Affiliated Hospital of Zhejiang University School of Medicine, Hangzhou, China; ^2^Department of Neurology, The Second Affiliated Hospital of Zhejiang University School of Medicine, Hangzhou, China

**Keywords:** Alzheimer’s disease, A/T/N system, spontaneous neuronal activity, functional magnetic resonance imaging, fractional amplitude of low-frequency fluctuation, cerebrospinal fluid

## Abstract

**Background**: The National Institute on Aging-Alzheimer’s Association (NIA-AA) has proposed a biological definition of Alzheimer’s disease (AD): individuals with both abnormal amyloid and tau biomarkers (A+T+) would be defined as AD. It remains unclear why different cognitive status is present in subjects with biological AD. Resting-state functional magnetic resonance imaging (rsfMRI) has provided an opportunity to reveal the brain activity patterns in a biologically-defined AD cohort. Accordingly, we aimed to investigate distinct brain activity patterns in subjects with existed AD pathology but in the different cognitive stages.

**Method**: We selected individuals with AD pathology (A+T+) and healthy controls (HC, A−T−) based on the cerebrospinal fluid (CSF) biomarkers. According to the cognitive stage, we divided the A+T+ cohort into three groups: (1) preclinical AD; (2) prodromal AD; and (3) AD with dementia (d-AD). We compared spontaneous brain activity measured by a fractional amplitude of low-frequency fluctuation (fALFF) approach among four groups.

**Results**: The analysis of covariance (ANCOVA) results showed significant differences in fALFF in the posterior cingulate cortex/precuneus (PCC/PCu). Further, compared to HC, we found increased fALFF values in the right inferior frontal gyrus (IFG) in the preclinical AD stage, whereas prodromal AD patients showed reduced fALFF in the bilateral precuneus, right middle frontal gyrus (MFG), right precentral gyrus, and postcentral gyrus. Within the d-AD group, both hyperactivity (right fusiform gyrus, right parahippocampal gyrus (PHG)/hippocampus, and inferior temporal gyrus) and hypoactivity (bilateral precuneus, left posterior cingulate cortex, left cuneus and superior occipital gyrus) were detected.

**Conclusion**: We found the distinct brain activity patterns in different cognitive stages among the subjects defined as AD biologically. Our findings may be helpful in understanding mechanisms leading to cognitive changes in the AD pathophysiological process.

## Introduction

Alzheimer’s disease (AD) remains the most common cause of dementia and is characterized by cognitive impairment, particularly in salient amnestic symptoms (Rogan and Lippa, 2002; Mucke, 2009). However, AD-related pathological processes, mainly including intracellular neurofibril tangles (NFTs) and extracellular amyloid deposition, begin years even decades before the appearance of clinical symptoms. Recently, the National Institute on Aging-Alzheimer’s Association (NIA-AA) has considered that amyloid (A) and tau (T) biomarkers indicate specific neuropathologic changes of AD, and proposed a new biological definition: individuals with biomarker profile “A+T+” (both abnormal A and T biomarkers) would be defined as AD (Jack et al., 2018). On the one hand, this definition could be helpful to exclude other diseases relating to cognitive impairment; on the other hand, this definition may detect subjects with AD-associated pathology in an early stage. However, different cognitive stages are present in the population among the people defined as biological AD. Individuals with biomarker profile “A+T+” could be subdivided into three categories: (1) preclinical AD (A+T+, cognitively unimpaired); (2) prodromal AD [A+T+, mild cognitive impairment (MCI)]; and (3) AD with dementia (A+T+, dementia; Jack et al., 2018). It remains unclear why individuals with existed AD pathology show different cognitive statuses.

Resting-state functional magnetic resonance imaging (rsfMRI) has revealed different patterns of spontaneous brain activity in patients with AD and MCI in a sensitive way. Notably, findings from several studies were contradictory. For example, some studies have found significantly decreased amplitude of low-frequency fluctuation (ALFF) in parahippocampal gyrus (PHG), superior temporal gyrus (STG) and postcentral gyrus in AD patients (Liu et al., 2014; Cha et al., 2015; Zheng et al., 2018), whereas some have reported increased ALFF in these regions (Wang et al., 2011). Inconsistent results could also be found in studies focus on patients with MCI (Wang et al., 2011; Xi et al., 2012) or amnestic MCI (aMCI; Han et al., 2011; Cai et al., 2017; Pan et al., 2017). We considered that the heterogeneity of patients, particularly suspected non-Alzheimer’s pathology, may confound neuroimaging results to some extent. Therefore, combing the AD biological definition described above and rsfMRI measures may be helpful to find more reliable brain activity change due to AD.

In the current study, we aimed to investigate different brain activity patterns among subjects labeled as preclinical AD, prodromal AD and AD with dementia (d-AD). We measured spontaneous brain activity *via* a fractional ALFF (fALFF) approach (Zou et al., 2008). This approach could improve the sensitivity and specificity in detecting regional spontaneous brain activity compared to ALFF (Zou et al., 2008), and has been widely used in major depressive disorder (Cheng et al., 2017; Qiu et al., 2019), schizophrenia (Hoptman et al., 2010; Zhang et al., 2018), Parkinson’s disease (Tang et al., 2017; Guo et al., 2018) and other neuropsychiatric disorders.

## Materials and Methods

### Participants

Data used in the preparation of this article were obtained from the Alzheimer’s Disease Neuroimaging Initiative (ADNI) database^1^. The ADNI was launched in 2003 by the National Institute on Aging (NIA), the National Institute of Biomedical Imaging and Bioengineering (NIBIB), the Food and Drug Administration (FDA), private pharmaceutical companies, and non-profit organizations, as a $60 million, 5-year public-private partnership. The primary goal of ADNI has been to test whether serial MRI, positron emission tomography (PET), other biological markers, and clinical and neuropsychological assessment can be combined to measure the progression of MCI and early AD. Determination of sensitive and specific markers of very early AD progression is intended to aid researchers and clinicians in developing new treatments and monitor their effectiveness, as well as lessen the time and cost of clinical trials.

Every subject has cerebrospinal fluid (CSF) biomarkers, high resolution T1-weighted 3D images and rsfMRI images approximately at the same time. Based on previous CSF biomarkers cut-off value, amyloid-β_42_ (Aβ_42_) and phosphorylated tau (p-tau) levels were considered abnormal if ≤192 ng/L and ≥23 ng/L, respectively (Shaw et al., 2009). Finally, we defined 11 cognitively unimpaired subjects with biomarker profile “A−T−” as healthy controls (HC). According to cognitive performance, we identified 75 individuals labeled as “A+T+,” and classified into three groups: 22 preclinical AD (cognitively unimpaired), 33 prodromal AD (MCI), and 20 d-AD (dementia).

### CSF Samples and Quantification

CSF data were downloaded from the ADNI. Levels of Aβ_42_, t-tau and p-tau were measured from CSF samples, which were obtained using the standardized ADNI protocol, as previously described (Shaw et al., 2011).

### Image Acquisition

All participants were scanned using a 3.0-Tesla MRI scanner. The structural image was acquired using a 3D T1-weighted sequence with the following parameters: axial orientation, 2,300 ms TR, 2.98 ms TE, 900 ms TI, 9° FA, each 1.2 mm in thickness. The rsfMRI scans were obtained using an echo-planar imaging sequence with the following parameters: 140 time points; TR = 3,000 ms; TE = 30 ms; flip angle = 80°; number of slices = 48; slice thickness = 3.3 mm; spatial resolution = 3.31 × 3.31 × 3.31 mm^3^; matrix = 64 × 64. According to the human scan protocol of the ADNI database, all subjects should keep their eyes open with fixation (focus on a point on the mirror) during the entire fMRI scan.

### Imaging Data Preprocessing and fALFF Calculation

We pre-processed rsfMRI data using the Data Processing and Analysis for (Resting-State) Brain Imaging, DPABI^2^ (Yan et al., 2016) with Statistical Parametric Mapping 12, SPM12^3^ on the MATLAB platform (MathWorks, Natick, MA, USA). The first 10 image volumes of functional images were discarded for the signal equilibrium and subject’s adaptation to the scanning noise. The remaining 130 images were corrected for timing differences between each slice and head motion (Friston 24 parameter). The structural MR images were segmented into gray matter (GM), white matter (WM) and CSF using SPM12. Subsequently, based on rigid-body transformation, the T1-weighted image was co-registered to the mean rsfMRI image and spatially normalized to the Montreal Neurological Institute (MNI) stereotactic space, then re-sampled them into of 3 × 3 × 3 mm^3^ cubic voxels. We removed linear trends and regressed out covariates, including Friston 24 head motion parameters, a signal of WM and CSF signal. The functional images were spatially smoothed with a Gaussian kernel of 6 × 6 × 6 mm^3^ full widths at half maximum to decrease spatial noise. Finally, data were scrubbed to further reduce motion-related artifacts by using a frame-wise displacement (FD) threshold of 0.5, with which “bad” time points were deleted. In this study, we excluded two preclinical AD group, one prodromal AD, and 6 d-AD due to excessive head motion (>3.0 mm maximum displacement in any of the x, y, or z directions or >3.0° of any angular motion).

The preprocessed functional images were imported into the DPABI toolbox (Yan et al., 2016) to calculate fALFF. In particular, the time series were first converted to a frequency domain with a fast Fourier transform, and the power spectrum was obtained. The square root of the power spectrum was computed at each frequency of the power spectrum, and the averaged square root was obtained across 0.01–0.08 Hz at each voxel. Finally, the fALFF was calculated as the ratio of the low-frequency power spectrum to the power spectrum of the entire frequency range (Zou et al., 2008; Yan et al., 2016).

### Statistical Analysis

Regarding demographic data, we used the analysis of variance (ANOVA) to compare the age, education, neuropsychological scales and CSF biomarkers among four groups; subsequently, we performed the *post hoc* two-sample *t*-test. The Chi-square test was used for gender distribution difference assessment (*p* < 0.05).

Based on the DPABI toolbox, we used the analysis of covariance (ANCOVA) to detect the whole-brain fALFF differences among HC, preclinical AD, prodromal AD, and d-AD groups, corrected by age, gender, education and mean FD. The clusters with significant between-group differences were defined as ROIs, and mean fALFF values were extracted from these ROIs. We performed a *post hoc* analysis between each group. To test the clinical significance, We then correlated mean fALFF values from ROIs with neuropsychological scales and CSF biomarkers. *Post hoc* tests and correlation analyses were corrected for multiple comparisons by Bonferroni correction (*p* < 0.05/6 and *p* < 0.05/5 respectively).

Further, to explore the differences in fALFF between the HC and other groups, we performed a second-level random-effect two-sample *t*-test (controlling mean age, education, gender, and FD) on the individual normalized fALFF maps in a voxel-wise manner.

The Gaussian random field (GRF) correction was used to correct for multiple comparisons. The statistical threshold was set at *p* < 0.01 with a cluster-level *p* < 0.05 (two-tailed) in DPABI^4^. GRF-based methods could control the family-wise error rate (FWER) of testing multiple hypotheses by assessing whether the test statistic or the spatial extent of clusters exceeding a cluster-defining threshold is large by chance (Bansal and Peterson, 2018; Yang et al., 2019). Notably, GRF correction considers spatial smoothness. Thus it could improve the sensitivity of detecting large activation clusters (Friston et al., 1994; Worsley et al., 1996).

Considering all HC were female, we also performed a supplementary analysis only with the females of each group.

## Results

### Demographic and Clinical Characteristics

Table 1 summarized the details of demographics, CSF biomarker levels (Aβ_42_, p-tau and t-tau), and corresponding neuropsychological tests. Three groups (preclinical AD, prodromal AD, d-AD) did not differ from HC concerning age and education (age: over-all ANOVA across the four groups; *F* = 2.181, *P* = 0.098; comparison of each group vs. HC using *t*-tests: preclinical AD vs. HC: *P* > 0.779; prodromal AD vs. HC: *P* > 0.108; d-AD vs. HC: *P* > 0.952; education: over-all ANOVA across the four groups; *F* = 0.698; *P* = 0.556; comparison of three groups vs. HC using *t*-tests: preclinical AD vs. HC: *P* > 0.954; prodromal AD vs. HC: *P* > 0.495; d-AD vs. HC: *P* > 0.684). However, there was difference on the distribution of gender between three groups and the HC (overall Fisher’s exact test: *P* = 0.002; Fisher’s exact test of comparisons between three groups and HC: preclinical AD vs. HC: *P* = 0.012; prodromal AD vs. HC: *P* = 0.001; d-AD vs. HC: *P* = 0.001).

**Table 1:** Demographics, neuropsychological and cerebrospinal fluids (CSF) assessments of the four groups.

	Normal control	Preclinical AD	Prodromal AD	d-AD	*p*-value
*N*	11	20	32	14	-
Mean age	75.36 ± 8.22	74.62 ± 6.11	71.48 ± 6.20	75.51 ± 4.06	0.098^a^
Gender (M/F)	0/11	9/11	19/13	9/5	0.002^b^
Education	16.09 ± 6.42	16.15 ± 2.28	16.72 ± 2.29	15.57 ± 2.87	0.556^a^
MMSE	29.46 ± 1.04	28.60 ± 1.96	27.88 ± 1.79	21.36 ± 3.65	<0.001^a^
TMT-A	32.64 ± 9.22	35.72 ± 8.41^c^	34.84 ± 16.19	72.71 ± 43.67	<0.001
TMT-B	61.82 ± 19.90	88.50 ± 39.14^c^	104.03 ± 60.51	198.14 ± 77.46	<0.001
CSF biomarkers					
Aβ_42_	230.73 ± 27.59	150.63 ± 25.54	138.80 ± 22.91	125.69 ± 19.27	<0.001^a^
p-tau	16.76 ± 4.19	44.60 ± 14.81	54.18 ± 25.00	56.54 ± 30.29	<0.001^a^
t-tau	49.65 ± 16.54	81.17 ± 35.61	114.34 ± 56.07^d^	136.44 ± 91.87	<0.001^a^

*Data are presented as mean ± SD. AD, Alzheimer’s disease; d-AD, AD with dementia; MMSE, Mini-Mental State Examination; TMT-A, Trail Making Test A; TMT-B, Trail Making Test B; Aβ_42_, amyloid-beta_42_; p-tau, phosphorylated tau; t-tau, total tau. ^a^The *p*-value was obtained by one-way analysis of variance (ANOVA). ^b^The *p*-value was obtained by a two-tail Fisher’s exact test. ^c^There were two missing values in the preclinical AD group. ^d^There were two missing values in the prodromal AD group*.

### fALFF Analyses

Tables s 2, 3 summarized the details of the brain regions with a difference in spontaneous activity.

**Table 2:** Analysis of covariance (ANCOVA) results with age, gender, education and the mean FD as covariates across the four groups.

	Brain region	Peak MNI coordinate			Peak intensity	Number of voxels
		X	Y	Z		
Without GM correction	PCC/PCu	−9	−51	27	7.223	75
GM correction	PCC/PCu	6	−57	36	7.403	68

*fALFF, fractional amplitude of low-frequency fluctuation; GM, gray matter; FD, frame-wise displacement; PCC/PCu, posterior cingulate cortex/Precuneus; MNI, Montreal Neurological Institute. The statistical threshold was set at *p* < 0.01 with a cluster-level *p* < 0.05 (two-tailed, GRF corrected)*.

**Table 3:** Brain areas showing significant fALFF differences between HC and other groups (controlled for age, education, gender and head motion).

	Brain region	Peak MNI coordinate			Peak intensity	Number of voxels
		X	Y	Z		
Preclinical AD	IFG	51	21	−3	4.399	63
Prodromal AD	PCC/PCu	9	−51	42	−3.999	136
Prodromal AD	MFG	33	−6	54	−4.553	78
d-AD	PHG	36	−33	−21	6.047	133
d-AD	PCC/PCu	−15	−66	30	−6.110	144

*HC, healthy control; d-AD, AD with dementia; fALFF, fractional amplitude of low-frequency fluctuations; IFG, inferior frontal gyrus; PHG, parahippocampal gyrus; MFG, middle frontal gyrus; PCC/PCu, posterior cingulate cortex/Precuneus; MNI, Montreal Neurological Institute. The statistical threshold was set at *p* < 0.01 with a cluster-level *p* < 0.05 (two-tailed, GRF corrected)*.

**Figure 1:**
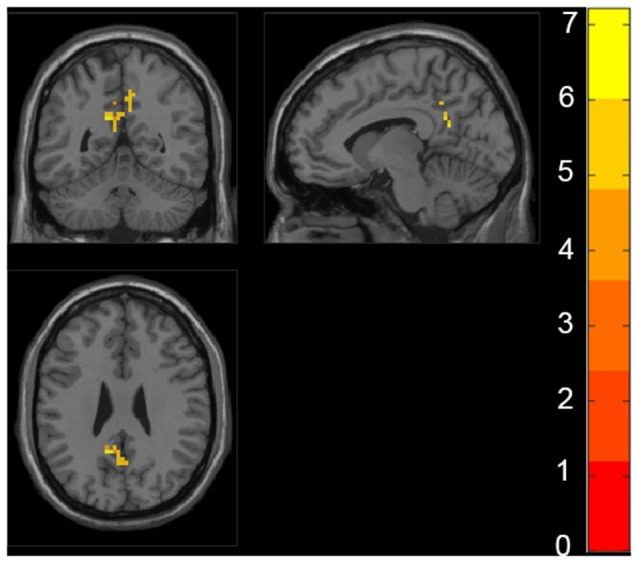
Regions with significant differences in fractional amplitude low-frequency fluctuations (fALFF) among healthy controls (HC), preclinical Alzheimer’s disease (AD), prodromal AD, and d-AD. The results were obtained by analysis of covariance (ANCOVA) analysis adjusted with mean age, gender, education and mean frame-wise displacement [FD; *P* < 0.01, cluster level < 0.05, two-tailed, gaussian random field (GRF) correction].

The ANCOVA revealed a significant difference in the bilateral posterior cingulate cortex/Precuneus (PCC/PCu) among four groups adjusted with mean age, gender, education and mean FD (*P* < 0.01, cluster level < 0.05, GRF correction; Figure 1). *Post hoc* analyses only found significant differences of fALFF value in PCC/PCu between HC and the rest of the three groups at the level of *p* < 0.05/6 after Bonferroni correction. There was no significant difference between other groups (Supplementary Table S1, Supplementary Figure S1).

**Figure 2:**
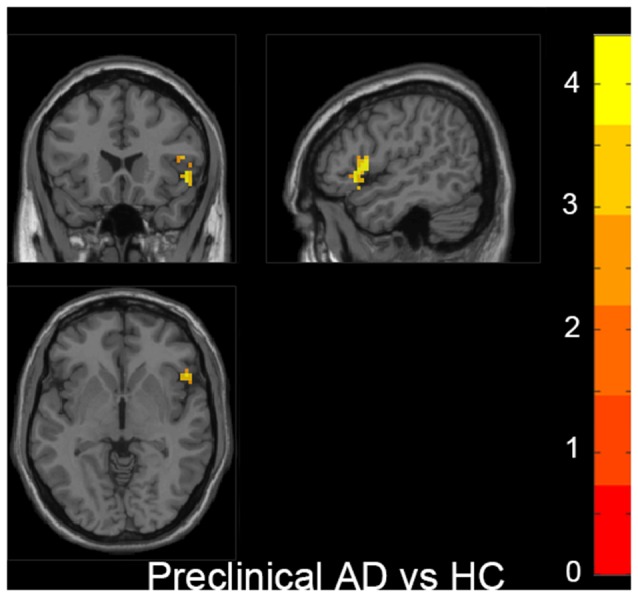
Increased fALFF was found in the right inferior frontal gyrus (IFG) in the preclinical AD group compared to HC. The statistical threshold was set at *p* < 0.01 with a cluster-level *p* < 0.05 (two-tailed, GRF corrected). The left hemisphere of the brain corresponds to the left side of the image.

**Figure 3:**
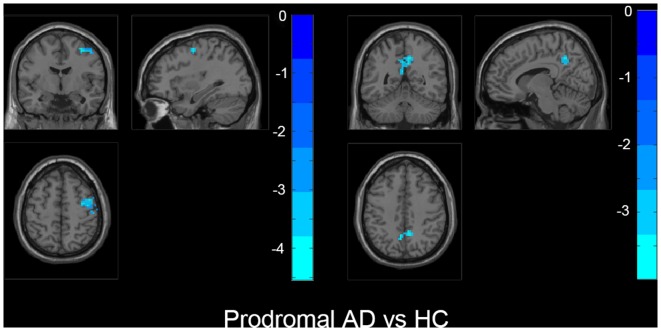
The prodromal AD group showed decreased fALFF in bilateral precuneus, right middle frontal gyrus (MFG), right precentral gyrus, and postcentral gyrus than HC. The statistical threshold was set at *p* < 0.01 with a cluster-level *p* < 0.05 (two-tailed, GRF corrected). The left hemisphere of the brain corresponds to the left side of the image.

**Figure 4:**
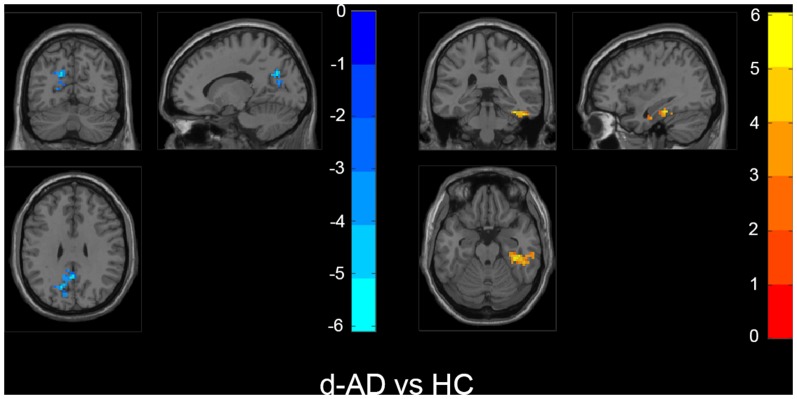
Within the d-AD group (compared to HC), regions with increased fALFF mainly included right fusiform gyrus, right parahippocampal gurus, right hippocampus and inferior temporal gyrus, whereas bilateral PCu, left PCC, left cuneus, and superior occipital gyrus had decreased fALFF values. The statistical threshold was set at *p* < 0.01 with a cluster-level *p* < 0.05 (two-tailed, GRF corrected). The left hemisphere of the brain corresponds to the left side of the image.

Also, we evaluated the fALFF difference between HC and other groups. We found that the preclinical AD group has increased fALFF values in the right inferior frontal gyrus (IFG) than HC (Figure 2). The prodromal AD patients showed reduced fALFF in the bilateral PCu, right middle frontal gyrus (MFG), right precentral gyrus and postcentral gyrus (Figure 3). Within the d-AD group, regions with decreased fALFF mainly included bilateral PCu, left PCC, left cuneus, and superior occipital gyrus, whereas right fusiform gyrus, right PHG, right hippocampus (HP) and inferior temporal gyrus had increased fALFF values (Figure 4). While taking regional GM volume as covariates, most of the results were similar to those without correction (Supplementary Table S2).

Similar results were obtained in the sample only with the females (Supplementary Table S3, Supplementary Figure S2).

### Correlations Between Neuropsychological Tests and fALFF Values

We correlated fALFF values in ROIs with neuropsychological scales (MMSE score). The PCC/Pcu showed significant correlations with Mini-Mental State Examination (MMSE), Trail Making Test-A (TMT-A), Trail Making Test-B (TMT-B), CSF Aβ_42_ and p-tau (Table 4).

**Table 4:** Correlation between fALFF values in PCC/PCu, neuropsychological scales, and CSF biomarkers.

	MMSE		TMT-A		TMT-B		Aβ_42_		p-tau	
	*r*	*p*	*r*	*p*	*r*	*p*	*r*	*p*	*r*	*p*
Without GM correction	0.301	0.008^b^	−0.234	0.043^a^	−0.346	0.002^ab^	0.486	<0.001^ab^	−0.280	0.014^a^
GM correction	0.224	0.050^a^	−0.179	0.125	−0.284	0.013^a^	0.454	<0.001^ab^	−0.253	0.026^a^

*fALFF, fractional amplitude of low-frequency fluctuations; PCC/PCu, Posterior cingulate cortex/Precuneus; GM, gray matter; MMSE, Mini-Mental State Examination; TMT-A, Trail Making Test A; TMT-B, Trail Making Test B; Aβ_42_, amyloid-beta_42_; p-tau, phosphorylated-tau; t-tau, total tau. Pearson’s correlation coefficient was reported. ^a^Means significance level of *P* < 0.05, ^b^represents significance level of *P* < 0.05/5 (with Bonferroni correction)*.

### fALFF Analyses With GM Volume as Covariates

We performed voxel-based morphometry (VBM) analysis to reveal the GM volume differences among the three groups. The results showed the most significant differences in the frontal, temporal, and parietal regions. While taking regional GM volume as covariates, we found that the increased fALFF in the IFG within the preclinical group could not survive, but other results were similar to those without correction.

## Discussion

To our knowledge, we employed rsfMRI to investigate potential AD-related brain activity change based on the AD biological diagnosis criteria for the first time. We found significant differences of fALFF in bilateral PCC/PCu among HC, preclinical AD, prodromal AD, and d-AD. Compared to HC, the rest of the three groups showed significant fALFF differences in several regions, including the IFG, the PCC/PCu, the PHG/HP, cuneus, MFG, superior occipital gyrus, and fusiform gyrus. Most regions have been reported in previous AD or MCI studies. We considered that the regions found in our study were more associated with pathology because of following two points: on the one hand, we only included subjects under the impact of AD pathology based on the CSF biomarkers; on the other hand, only the subjects with normal cognition and biomarkers would be selected as controls. Thus, these could reduce the influences of non-Alzheimer’s pathologic change.

Regarding brain activity patterns, three aspects of the results merit particular attention. First, we found significant differences of fALFF in PCC/PCu among four groups, which were related to neuropsychological tests and CSF biomarkers. Also, both the prodromal AD and the d-AD group showed reduced fALFF than HC in these regions. Consistently, previous studies reported that PCC/PCu had structural or functional abnormalities in patients with AD and MCI, such as cortical thinning (Lerch et al., 2005; Singh et al., 2006; He et al., 2008; Dickerson et al., 2009) and metabolic disruptions (de Leon et al., 2001; Volkow et al., 2002; Yokoi et al., 2018). Both the PCC and PCu are essential components of the default mode network (DMN) and are considered as brain network hub regions which have widespread connections to other areas (Cavanna and Trimble, 2006; Zhang and Li, 2012; Leech and Sharp, 2014; Utevsky et al., 2014). Pathologically, the PCC/PCu is involved in Braak’s stage IV and V, highly relating to the emergence of initial symptoms and full development of AD, respectively. Using PET data, Hoenig et al. (2018) found that tau pathology network (TPN) overlapped with the DMN and the frontal control network, and the peaks of tau within TPNs included the PCu, PCC, and PHG. Tau retention in the PCC/PCu could result in the disruption of brain networks crucial for cognitive function. Thus, clinical symptoms appear first in the prodromal AD stage. Previous studies focusing on the AD spectrum without application of biomarkers have also reported that bilateral PCC showed significant differences in brain activity (Yang et al., 2018, 2019). Thus, the hypoactivity in PCC/PCu may be a potential imaging biomarker that reflects early cerebral change under AD pathology.

Second, within the preclinical AD group, increased fALFF values in the IFG were detected compared to HC. Functionally, the frontal lobe participates in episodic memory (Matthews, 2015) and is associated with maintenance of memory (de Chastelaine et al., 2011; McLaren et al., 2012). Under the impact of amyloid and tau pathology, we interpreted the hyperactivity in the IFG as compensational effects so that the individuals could maintain normal cognitive performance for a period. Recently, Lin et al. (2017) have found that higher activity and stronger functional connectivity in the IFG and insula may protect memory performance against AD-associated pathology. Moreover, the higher BOLD signal in the IFG during the encoding process also has been found in participants classified into a successful group according to performance in the memory task (Pudas et al., 2013). Our finding supported further that the frontal lobe might be one of the key regions to keep cognitive function in individuals with existed AD pathology.

Third, the d-AD group showed increased fALFF than HC in the right PHG/HP, right fusiform gyrus and inferior temporal gyrus. Neuronal hyperactivity has been an increasingly observed phenomenon in the AD pathological cascade (Palop et al., 2007; Bero et al., 2011; Busche and Konnerth, 2015; Palop and Mucke, 2016). Some animal studies have found hyperactive neurons in the HP and hyperactivity might be linked to dysfunction of the HP (Abramowski et al., 2008; Palop and Mucke, 2010; DeVos et al., 2013; Krüger and Mandelkow, 2016; Wu et al., 2016). Furthermore, hippocampal hyperactivity is related to amyloid deposition and pathologic tau accumulation in these animal models (Abramowski et al., 2008; Palop and Mucke, 2010; DeVos et al., 2013; Krüger and Mandelkow, 2016; Wu et al., 2016). Human neuroimaging studies have linked hippocampal hyperactivity to amyloid accumulation (Huijbers et al., 2015), apolipoprotein E4 (APOE4; Bookheimer et al., 2000; Johnson et al., 2006; Filippini et al., 2011; Tran et al., 2017) and progression to dementia (Huijbers et al., 2015). The increased hippocampal activity might be a pathological response, or compensation, or both. However, in the present study, d-AD was suggested as a late stage in Alzheimer’s continuum, which has severe cognitive symptoms. Thus, we interpreted that the increased activity in this stage might reflect a pathological response that results in cognitive deficits.

Also, there were some limitations. First, our sample size was relatively small, and all HC were female. Few subjects have both the rsfMRI and CSF biomarkers, although the ADNI datasets have a large sample size in AD patients, MCI patients and people with normal cognition. Despite the small sample size, our preliminary study would be helpful for a better understanding of pathophysiological mechanisms in individuals with existed AD pathology but with different cognitive functions. It may provide a new insight for potential AD-related brain activity change. Another limitation is the lack of PET data. According to the research framework, both CSF Aβ_42_ and amyloid PET are suggested as A biomarker, whereas CSF p-tau and tau PET as T biomarkers (Jack et al., 2018). In this study, CSF biomarkers were used to confirm individuals’ biomarker profile because of a smaller number of tau PET. It is widely-accepted that CSF Aβ_42_ and p-tau level could reflect the pathologic state associated with amyloid plaque formation and paired helical filament (PHF) tau formation. CSF biomarkers are not the measures of amyloid plaque load and pathologic tau deposits as PET is (Jack et al., 2018). Further large sample studies combining PET and MRI data would be beneficial for finding more precise mechanisms underlying cognitive symptoms.

## Conclusion

We found different patterns of brain activity in different cognitive stages among the subjects defined as AD biologically. Our findings would be helpful in understanding mechanisms leading to cognitive changes in the AD pathophysiological process.

## Data Availability Statement

The datasets generated and/or analyzed during the current study are available in the ADNI study. More details in www.adni-info.org.

## Author Contributions

QZ designed the study and wrote the first draft of the manuscript. XL analyzed the MRI data and wrote the protocol. QZ, KL, and XL collected clinical and MRI data. SW, RZ, HH, PH, YJ, XX, JX, CW, JZ, and MZ assisted with study design and interpretation of findings. All authors have contributed to, read and approved the final manuscript.

## Conflict of Interest

The authors declare that the research was conducted in the absence of any commercial or financial relationships that could be construed as a potential conflict of interest.

## Footnotes

^1^http://adni.loni.usc.edu/

^2^http://rfmri.org/dpabi

^3^http://www.fil.ion.ucl.ac.uk/spm/

^4^http://rfmri.org/dpabi

## References

[B1] Abramowski D. Wiederhold K. H. Furrer U. Jaton A. L. Neuenschwander A. Runser M. J.. (2008). Dynamics of A beta turnover and deposition in different beta-amyloid precursor protein transgenic mouse models following gamma-secretase inhibition. J. Pharmacol. Exp. Ther. 327, 411–424. 10.1124/jpet.108.140327 18687920

[B2] Bansal R. Peterson B. S. (2018). Cluster-level statistical inference in fMRI datasets: the unexpected behavior of random fields in high dimensions. Magn. Reson. Imaging 49, 101–115. 10.1016/j.mri.2018.01.004 29408478 PMC5991838

[B3] Bero A. W. Yan P. Roh J. H. Cirrito J. R. Stewart F. R. Raichle M. E.. (2011). Neuronal activity regulates the regional vulnerability to amyloid-α deposition. Nat. Neurosci. 14, 750–756. 10.1038/nn.2801 21532579 PMC3102784

[B4] Bookheimer S. Y. Strojwas M. H. Cohen M. S. Saunders A. M. Pericak-Vance M. A. Mazziotta J. C.. (2000). Patterns of brain activation in people at risk for Alzheimer’s disease. N. Engl. J. Med. 343, 450–456. 10.1056/NEJM200008173430701 10944562 PMC2831477

[B5] Busche M. A. Konnerth A. (2015). Neuronal hyperactivity–A key defect in Alzheimer–s disease? Bioessays 37, 624–632. 10.1002/bies.201500004 25773221

[B6] Cai S. Chong T. Peng Y. Shen W. Li J. von Deneen K. M.. (2017). Altered functional brain networks in amnestic mild cognitive impairment: a resting-state fMRI study. Brain Imaging Behav. 11, 619–631. 10.1007/s11682-016-9539-0 26972578

[B7] Cavanna A. E. Trimble M. R. (2006). The precuneus: a review of its functional anatomy and behavioural correlates. Brain 129, 564–583. 10.1093/brain/awl004 16399806

[B8] Cha J. Hwang J. M. Jo H. J. Seo S. W. Na D. L. Lee J. M. (2015). Assessment of functional characteristics of amnestic mild cognitive impairment and Alzheimer’s disease using various methods of resting-state FMRI analysis. Biomed Res. Int. 2015:907464. 10.1155/2015/907464 26180816 PMC4477185

[B9] Cheng Y. Xu J. Arnone D. Nie B. Yu H. Jiang H.. (2017). Resting-state brain alteration after a single dose of SSRI administration predicts 8-week remission of patients with major depressive disorder. Psychol. Med. 47, 438–450. 10.1017/s0033291716002440 27697079

[B10] de Chastelaine M. Wang T. H. Minton B. Muftuler L. T. Rugg M. D. (2011). The effects of age, memory performance and callosal integrity on the neural correlates of successful associative encoding. Cereb. Cortex 21, 2166–2176. 10.1093/cercor/bhq294 21282317 PMC3155606

[B11] de Leon M. J. Convit A. Wolf O. T. Tarshish C. Y. DeSanti S. Rusinek H.. (2001). Prediction of cognitive decline in normal elderly subjects with 2-[^18^F]fluoro-2-deoxy-D-glucose/poitron-emission tomography (FDG/PET). Proc. Natl. Acad. Sci U S A 98, 10966–10971. 10.1073/pnas.191044198 11526211 PMC58582

[B12] DeVos S. L. Goncharoff D. K. Chen G. Kebodeaux C. S. Yamada K. Stewart F. R.. (2013). Antisense reduction of tau in adult mice protects against seizures. J. Neurosci. 33, 12887–12897. 10.1523/jneurosci.2107-13.2013 23904623 PMC3728694

[B13] Dickerson B. C. Bakkour A. Salat D. H. Feczko E. Pacheco J. Greve D. N.. (2009). The cortical signature of Alzheimer’s disease: regionally specific cortical thinning relates to symptom severity in very mild to mild AD dementia and is detectable in asymptomatic amyloid-positive individuals. Cereb. Cortex 19, 497–510. 10.1093/cercor/bhn113 18632739 PMC2638813

[B14] Filippini N. Ebmeier K. P. MacIntosh B. J. Trachtenberg A. J. Frisoni G. B. Wilcock G. K.. (2011). Differential effects of the APOE genotype on brain function across the lifespan. Neuroimage 54, 602–610. 10.1016/j.neuroimage.2010.08.009 20705142

[B15] Friston K. J. Worsley K. J. Frackowiak R. S. Mazziotta J. C. Evans A. C. (1994). Assessing the significance of focal activations using their spatial extent. Hum. Brain Mapp. 1, 210–220. 10.1002/hbm.460010306 24578041

[B16] Guo T. Guan X. J. Zeng Q. L. Xuan M. Gu Q. Q. Xu X. J.. (2018). Correlations between CSF proteins and spontaneous neuronal activity in Parkinson’s disease. Neurosci. Lett. 673, 61–66. 10.1016/j.neulet.2018.02.062 29501577

[B17] Han Y. Wang J. Zhao Z. Min B. Lu J. Li K.. (2011). Frequency-dependent changes in the amplitude of low-frequency fluctuations in amnestic mild cognitive impairment: a resting-state fMRI study. Neuroimage 55, 287–295. 10.1016/j.neuroimage.2010.11.059 21118724

[B18] He Y. Chen Z. Evans A. (2008). Structural insights into aberrant topological patterns of large-scale cortical networks in Alzheimer’s disease. J. Neurosci. 28, 4756–4766. 10.1523/JNEUROSCI.0141-08.2008 18448652 PMC6670444

[B19] Hoenig M. C. Bischof G. N. Seemiller J. Hammes J. Kukolja J. Onur O. A.. (2018). Networks of tau distribution in Alzheimer’s disease. Brain 141, 568–581. 10.1093/brain/awx353 29315361

[B20] Hoptman M. J. Zuo X. N. Butler P. D. Javitt D. C. D’Angelo D. Mauro C. J.. (2010). Amplitude of low-frequency oscillations in schizophrenia: a resting state fMRI study. Schizophr. Res. 117, 13–20. 10.1016/j.schres.2009.09.030 19854028 PMC2822110

[B21] Huijbers W. Mormino E. C. Schultz A. P. Wigman S. Ward A. M. Larvie M.. (2015). Amyloid-α deposition in mild cognitive impairment is associated with increased hippocampal activity, atrophy and clinical progression. Brain 138, 1023–1035. 10.1093/brain/awv007 25678559 PMC4438387

[B22] Jack C. R. Jr. Bennett D. A. Blennow K. Carrillo M. C. Dunn B. Haeberlein S. B.. (2018). NIA-AA research framework: toward a biological definition of Alzheimer’s disease. Alzheimers Dement. 14, 535–562. 10.1016/j.jalz.2018.02.018 29653606 PMC5958625

[B23] Johnson S. C. Schmitz T. W. Trivedi M. A. Ries M. L. Torgerson B. M. Carlsson C. M.. (2006). The influence of Alzheimer disease family history and apolipoprotein E epsilon4 on mesial temporal lobe activation. J. Neurosci. 26, 6069–6076. 10.1523/jneurosci.0959-06.2006 16738250 PMC2684824

[B24] Krüger L. Mandelkow E. M. (2016). Tau neurotoxicity and rescue in animal models of human Tauopathies. Curr. Opin. Neurobiol. 36, 52–58. 10.1016/j.conb.2015.09.004 26431808

[B25] Leech R. Sharp D. J. (2014). The role of the posterior cingulate cortex in cognition and disease. Brain 137, 12–32. 10.1093/brain/awt162 23869106 PMC3891440

[B26] Lerch J. P. Pruessner J. C. Zijdenbos A. Hampel H. Teipel S. J. Evans A. C. (2005). Focal decline of cortical thickness in Alzheimer’s disease identified by computational neuroanatomy. Cereb. Cortex 15, 995–1001. 10.1093/cercor/bhh200 15537673

[B27] Lin F. Ren P. Lo R. Y. Chapman B. P. Jacobs A. Baran T. M.. (2017). Insula and inferior frontal gyrus’ activities protect memory performance against Alzheimer’s disease pathology in old age. J. Alzheimers Dis. 55, 669–678. 10.3233/jad-160715 27716674 PMC5531269

[B28] Liu X. Wang S. Zhang X. Wang Z. Tian X. He Y. (2014). Abnormal amplitude of low-frequency fluctuations of intrinsic brain activity in Alzheimer’s disease. J. Alzheimers Dis. 40, 387–397. 10.3233/jad-131322 24473186

[B29] Matthews B. R. (2015). Memory dysfunction. Continuum 21, 613–626. 10.1212/01.CON.0000466656.59413.29 26039844 PMC4455839

[B30] McLaren D. G. Sreenivasan A. Diamond E. L. Mitchell M. B. Van Dijk K. R. Deluca A. N.. (2012). Tracking cognitive change over 24 weeks with longitudinal functional magnetic resonance imaging in Alzheimer’s disease. Neurodegener. Dis. 9, 176–186. 10.1159/000335876 22456451 PMC3369254

[B31] Mucke L. (2009). Neuroscience Alzheimer’s disease. Nature 461, 895–897. 10.1038/461895a 19829367

[B32] Palop J. J. Mucke L. (2010). Amyloid-beta-induced neuronal dysfunction in Alzheimer’s disease: from synapses toward neural networks. Nat. Neurosci. 13, 812–818. 10.1038/nn.2583 20581818 PMC3072750

[B33] Palop J. J. Mucke L. (2016). Network abnormalities and interneuron dysfunction in Alzheimer disease. Nat. Rev. Neurosci. 17, 777–792. 10.1038/nrn.2016.141 27829687 PMC8162106

[B34] Palop J. J. Chin J. Roberson E. D. Wang J. Thwin M. T. Bien-Ly N.. (2007). Aberrant excitatory neuronal activity and compensatory remodeling of inhibitory hippocampal circuits in mouse models of Alzheimer’s disease. Neuron 55, 697–711. 10.1016/j.neuron.2007.07.025 17785178 PMC8055171

[B35] Pan P. Zhu L. Yu T. Shi H. Zhang B. Qin R.. (2017). Aberrant spontaneous low-frequency brain activity in amnestic mild cognitive impairment: a meta-analysis of resting-state fMRI studies. Ageing Res. Rev. 35, 12–21. 10.1016/j.arr.2016.12.001 28017880

[B36] Pudas S. Persson J. Josefsson M. de Luna X. Nilsson L. G. Nyberg L. (2013). Brain characteristics of individuals resisting age-related cognitive decline over two decades. J. Neurosci. 33, 8668–8677. 10.1523/jneurosci.2900-12.2013 23678111 PMC6618845

[B37] Qiu H. T. Li X. K. Luo Q. H. Li Y. M. Zhou X. C. Cao H. L.. (2019). Alterations in patients with major depressive disorder before and after electroconvulsive therapy measured by fractional amplitude of low-frequency fluctuations (fALFF). J. Affect. Disord. 244, 92–99. 10.1016/j.jad.2018.10.099 30326347 PMC6239214

[B38] Rogan S. Lippa C. F. (2002). Alzheimer’s disease and other dementias: a review. Am. J. Alzheimers Dis. Other Demen. 17, 11–17. 10.1177/153331750201700106 11831415 PMC10833834

[B39] Shaw L. M. Vanderstichele H. Knapik-Czajka M. Clark C. M. Aisen P. S. Petersen R. C.. (2009). Cerebrospinal fluid biomarker signature in Alzheimer’s disease neuroimaging initiative subjects. Ann. Neurol. 65, 403–413. 10.1002/ana.21610 19296504 PMC2696350

[B40] Shaw L. M. Vanderstichele H. Knapik-Czajka M. Figurski M. Coart E. Blennow K.. (2011). Qualification of the analytical and clinical performance of CSF biomarker analyses in ADNI. Acta Neuropathol. 121, 597–609. 10.1007/s00401-011-0808-0 21311900 PMC3175107

[B41] Singh V. Chertkow H. Lerch J. P. Evans A. C. Dorr A. E. Kabani N. J. (2006). Spatial patterns of cortical thinning in mild cognitive impairment and Alzheimer’s disease. Brain 129, 2885–2893. 10.1093/brain/awl256 17008332

[B42] Tang Y. Meng L. Wan C. M. Liu Z. H. Liao W. H. Yan X. X.. (2017). Identifying the presence of Parkinson’s disease using low-frequency fluctuations in BOLD signals. Neurosci. Lett. 645, 1–6. 10.1016/j.neulet.2017.02.056 28249785

[B43] Tran T. T. Speck C. L. Pisupati A. Gallagher M. Bakker A. (2017). Increased hippocampal activation in ApoE-4 carriers and non-carriers with amnestic mild cognitive impairment. Neuroimage Clin. 13, 237–245. 10.1016/j.nicl.2016.12.002 28070483 PMC5217770

[B44] Utevsky A. V. Smith D. V. Huettel S. A. (2014). Precuneus is a functional core of the default-mode network. J. Neurosci. 34, 932–940. 10.1523/JNEUROSCI.4227-13.2014 24431451 PMC3891968

[B45] Volkow N. D. Zhu W. Felder C. A. Mueller K. Welsh T. F. Wang G. J.. (2002). Changes in brain functional homogeneity in subjects with Alzheimer’s disease. Psychiatry Res. 114, 39–50. 10.1016/s0925-4927(01)00130-5 11864808

[B46] Wang Z. Yan C. Zhao C. Qi Z. Zhou W. Lu J.. (2011). Spatial patterns of intrinsic brain activity in mild cognitive impairment and Alzheimer’s disease: a resting-state functional MRI study. Hum. Brain Mapp. 32, 1720–1740. 10.1002/hbm.21140 21077137 PMC6870362

[B47] Worsley K. J. Marrett S. Neelin P. Vandal A. C. Friston K. J. Evans A. C. (1996). A unified statistical approach for determining significant signals in images of cerebral activation. Hum. Brain Mapp. 4, 58–73. 10.1002/(SICI)1097-0193(1996)4:1<58::AID-HBM4>3.0.CO;2-O 20408186

[B48] Wu J. W. Hussaini S. A. Bastille I. M. Rodriguez G. A. Mrejeru A. Rilett K.. (2016). Neuronal activity enhances tau propagation and tau pathology *in vivo*. Nat. Neurosci. 19, 1085–1092. 10.1038/nn.4328 27322420 PMC4961585

[B49] Xi Q. Zhao X. Wang P. Guo Q. Jiang H. Cao X.. (2012). Spontaneous brain activity in mild cognitive impairment revealed by amplitude of low-frequency fluctuation analysis: a resting-state fMRI study. Radiol. Med. 117, 865–871. 10.1007/s11547-011-0780-8 22241376

[B50] Yan C. G. Wang X. D. Zuo X. N. Zang Y. F. (2016). DPABI: data processing & analysis for (Resting-State) brain imaging. Neuroinformatics 14, 339–351. 10.1007/s12021-016-9299-4 27075850

[B51] Yang L. Yan Y. Li Y. Hu X. Lu J. Chan P.. (2019). Frequency-dependent changes in fractional amplitude of low-frequency oscillations in Alzheimer’s disease: a resting-state fMRI study. Brain Imaging Behav. [Epub ahead of print]. 10.1007/s11682-019-00169-6 31478145

[B52] Yang L. Yan Y. Wang Y. Hu X. Lu J. Chan P.. (2018). Gradual disturbances of the amplitude of low-frequency fluctuations (ALFF) and fractional ALFF in Alzheimer spectrum. Front. Neurosci. 12:975. 10.3389/fnins.2018.00975 30618593 PMC6306691

[B53] Yokoi T. Watanabe H. Yamaguchi H. Bagarinao E. Masuda M. Imai K.. (2018). Involvement of the precuneus/posterior cingulate cortex is significant for the development of Alzheimer’s disease: a PET (THK5351, PiB) and resting fMRI study. Front. Aging Neurosci. 10:304. 10.3389/fnagi.2018.00304 30344488 PMC6182068

[B54] Zhang P. Li Y. L. Fan F. M. Li C. S. R. Luo X. G. Yang F. D.. (2018). Resting-state brain activity changes associated with tardive dyskinesia in patients with schizophrenia: fractional amplitude of low-frequency fluctuation decreased in the occipital lobe. Neuroscience 385, 237–245. 10.1016/j.neuroscience.2018.06.014 29909076

[B55] Zhang S. Li C. S. (2012). Functional connectivity mapping of the human precuneus by resting state fMRI. Neuroimage 59, 3548–3562. 10.1016/j.neuroimage.2011.11.023 22116037 PMC3288461

[B56] Zheng W. Su Z. Liu X. Zhang H. Han Y. Song H.. (2018). Modulation of functional activity and connectivity by acupuncture in patients with Alzheimer disease as measured by resting-state fMRI. PLoS One 13:e0196933. 10.1371/journal.pone.0196933 29763448 PMC5953467

[B57] Zou Q. H. Zhu C. Z. Yang Y. Zuo X. N. Long X. Y. Cao Q. J.. (2008). An improved approach to detection of amplitude of low-frequency fluctuation (ALFF) for resting-state fMRI: fractional ALFF. J. Neurosci. Methods 172, 137–141. 10.1016/j.jneumeth.2008.04.012 18501969 PMC3902859

