# Supplementary material for: Distinct Spontaneous Brain Activity Patterns in Different Biologically-Defined Alzheimer’s Disease Cognitive Stage: A Preliminary Study
Source: Front Aging Neurosci. 2019 Dec 17;11:350. doi: 10.3389/fnagi.2019.00350 (PMC6980867; doi:10.3389/fnagi.2019.00350)
Supplement: Supplementary file 1 [file Table_1.DOCX]

Supplementary Material

# Supplementary Data

**Supplementary Methods and Materials**

The biomarkers could reflect AD-associated pathology. Jack et al. have first presented us with a new classification method called the “A/T/N” system which divides AD biomarkers into categories of amyloid (A), tau (T), and neurodegeneration (N). This classification method does not rely on cognitive assessment but more objective biomarkers, so it can avoid being confused by cognitive reserve; also, A/T/N system can be applied to classification of healthy elderly, participants with cognitive impairment, or patients with dementia. In a recent research framework, the National Institute on Aging-Alzheimer’s Association (NIA-AA) has proposed a novel biological definition of the AD: individuals with abnormalities in both amyloid (A) and tau (T) biomarkers would be defined as “Alzheimer’s disease (AD)”. Moreover, individuals with only abnormal A biomarkers but with normal T biomarkers would be assigned the label “Alzheimer’s pathologic change”. The Alzheimer’s continuum consists of the AD and pathologic change.

# Supplementary Tables

**Supplementary Table 1.** The fALFF values extracted from the PCC/PCu in each group (controlled for age, education, gender and head motion).

|  | HC | Preclinical AD | Prodromal AD | d-AD | Post-hoc |
| --- | --- | --- | --- | --- | --- |
| fALFF | 1.24±0.56 | 0.35±0.67 | 0.34±0.69 | 0.03±0.43 | HC>Preclinical AD, Prodromal AD, d-AD |
| fALFF  (GM corrected) | 1.24±0.54 | 0.31±0.70 | 0.30±0.74 | 0.13±0.52 | HC>Preclinical AD, Prodromal AD, d-AD |

**Supplementary Table 2.** Brain areas showing significant fALFF differences between three groups and healthy controls (controlled for age, education, gender, head motion and GM).

| Group | Brain region | Peak MNI coordinate | | | Peak intensity | Number of voxels |
| --- | --- | --- | --- | --- | --- | --- |
|  |  | X | Y | Z |  |  |
| Prodromal AD | PCC/PCu | 6 | -54 | 36 | -3.959 | 126 |
| Prodromal AD | MFG | 33 | -6 | 54 | -4.519 | 78 |
| d-AD | PHG | 39 | -30 | -21 | 5.589 | 108 |
| d-AD | PCC/PCu | -15 | -66 | 30 | -5.287 | 57 |

fALFF, fractional amplitude of low-frequency ﬂuctuations; GM, gray matter; PHG, parahippocampal gyrus; MFG, middle frontal gyrus; PCC/PCu, posterior cingulated cortex/Precuneus; MNI, Montreal Neurological Institute.

The statistical threshold was set at p < 0.01 with a cluster level p < 0.05 (two-tailed, GRF corrected).

**Supplementary Table 3.** ANCOVA results with age, education and mean FD as covariates across the four groups (only with the females).

|  | Brain region | Peak MNI coordinate | | | Peak intensity | Number of voxels |
| --- | --- | --- | --- | --- | --- | --- |
|  |  | X | Y | Z |  |  |
| Without GM correction | PCC/PCu | -3 | -54 | 30 | 6.62 | 9 |
| GM correction | PCC/PCu | 6 | -51 | 33 | 6.59 | 3 |

fALFF, fractional amplitude of low-frequency fluctuation; GM, gray matter; FD, frame-wise displacement; PCu, Precuneus; MNI, Montreal Neurological Institute.

## Supplementary Figures


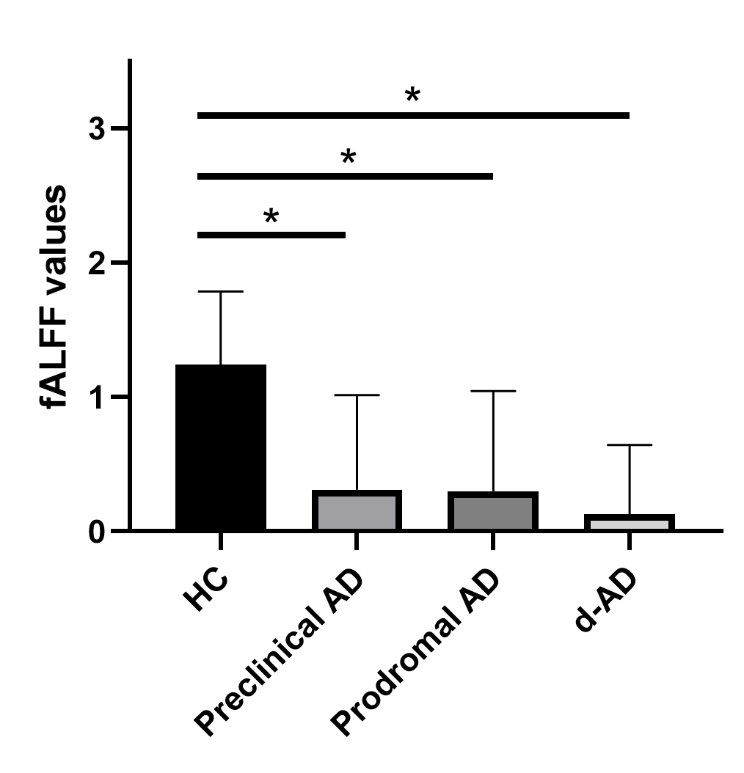


**Supplementary Fig 1.** A post hoc-test of main effect for group.

* means significance level of P < 0.05/6 (Bonferroni corrected).

fALFF, fractional amplitude of low-frequency fluctuation; HC, heathy control; AD, Alzheimer’s disease; d-AD, AD dementia.


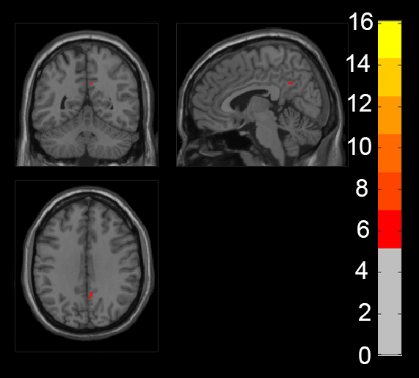


**Supplementary Fig 2.** Regions with significant differences in fractional amplitude low-frequency ﬂuctuations (fALFF) across the four groups (only with the females). The results were obtained by ANCOVA analysis adjusted with mean age, gender, education and mean FD (P < 0.01, cluster level < 0.05, two-tailed, GRF correction)
